# Parietal Contributions to Visual Working Memory Depend on Task Difficulty

**DOI:** 10.3389/fpsyt.2012.00081

**Published:** 2012-09-10

**Authors:** Kevin T. Jones, Marian E. Berryhill

**Affiliations:** ^1^Memory and Brain Laboratory, Department of Psychology, University of NevadaReno, NV, USA

**Keywords:** tDCS, PPC, working memory, task difficulty, individual differences

## Abstract

The nature of parietal contributions to working memory (WM) remain poorly understood but of considerable interest. We previously reported that posterior parietal damage selectively impaired WM probed by recognition (Berryhill and Olson, 2008a). Recent studies provided support using a neuromodulatory technique, transcranial direct current stimulation (tDCS) applied to the right parietal cortex (P4). These studies confirmed parietal involvement in WM because parietal tDCS altered WM performance: anodal current tDCS improved performance in a change detection task, and cathodal current tDCS impaired performance on a sequential presentation task. Here, we tested whether these complementary results were due to different degrees of parietal involvement as a function of WM task demands, WM task difficulty, and/or participants’ WM capacity. In Experiment 1, we applied cathodal and anodal tDCS to the right parietal cortex and tested participants on both previously used WM tasks. We observed an interaction between tDCS (anodal, cathodal), WM task difficulty, and participants’ WM capacity. When the WM task was difficult, parietal stimulation (anodal *or* cathodal) improved WM performance selectively in participants with high WM capacity. In the low WM capacity group, parietal stimulation (anodal *or* cathodal) impaired WM performance. These nearly equal and opposite effects were only observed when the WM task was challenging, as in the change detection task. Experiment 2 probed the interplay of WM task difficulty and WM capacity in a parametric manner by varying set size in the WM change detection task. Here, the effect of parietal stimulation (anodal or cathodal) on the high WM capacity group followed a linear function as WM task difficulty increased with set size. The low WM capacity participants were largely unaffected by tDCS. These findings provide evidence that parietal involvement in WM performance depends on both WM capacity and WM task demands. We discuss these findings in terms of alternative WM strategies employed by low and high WM capacity individuals. We speculate that low WM capacity individuals do not recruit the posterior parietal lobe for WM tasks as efficiently as high WM capacity individuals. Consequently, tDCS provides greater benefit to individuals with high WM capacity.

## Introduction

Keeping a running subtotal as we shop, remembering a new acquaintance’s name for a subsequent introduction, maintaining the distance of the car behind us as we switch lanes – these are examples of daily activities that rely on working memory (WM). WM serves as our mental workspace and as such it plays an essential role in cognition. Given this central role, cognitive researchers have devoted considerable efforts developing and refining theoretical models of WM (for reviews see Baddeley and Hitch, 1974; Cowan, 1993; Baddeley, 2000; Miyake et al., 2001; Oberauer, 2002; Curtis and D’Esposito, 2003; Cowan et al., 2005; Chein and Fiez, 2010). More recent work has focused on extending cognitive models to identify the neural correlates of WM, including the contributions of the inferior and superior parietal lobes comprising posterior parietal cortex (PPC; for reviews see Jonides et al., 1993; Cohen et al., 1997; Courtney et al., 1997; Ungerleider et al., 1998; Chein and Fiez, 2001; Munk et al., 2002; Pessoa et al., 2002; Linden et al., 2003; Sala et al., 2003; Olson and Berryhill, 2009; Brady et al., 2011). WM studies commonly identify PPC activations in functional magnetic resonance imaging (fMRI), yet only recently have these activations been functionally associated with WM rather than attention (Wager and Smith, 2003; Todd and Marois, 2004, 2005; Song and Jiang, 2006; Xu and Chun, 2006; Xu, 2007, 2009). Notably, activity in the intraparietal sulcus parametrically increases according to the number of items maintained in WM according to an individual’s WM capacity limit (Todd and Marois, 2004, 2005). These fMRI data point toward parietal involvement in WM maintenance, but converging evidence from neuropsychological patients is only partly consistent with this view. We found that patients with bilateral parietal damage were selectively impaired at blocks of WM trials probed by old/new recognition but not recall (Berryhill and Olson, 2008b). Yet, when recall and recognition WM trials were intermingled making the retrieval demands unpredictable these same patient participants could perform normally on recognition WM trials (Berryhill et al., 2011). Our conclusion was that under certain conditions the patients with bilateral parietal damage uniformly applied a recall strategy (e.g., in the unpredictable rather than the blocked WM task). We interpreted these data as indicative of PPC involvement in the strategic attentional refreshing of items in WM that were not subject to active verbal rehearsal (Berryhill et al., 2011). An important prediction that this view promotes is that when verbal rehearsal strategies are limited, the PPC is needed for accurate WM performance.

One complementary approach to the neuropsychological and neuroimaging described above is neuromodulatory. Here, we used transcranial direct current stimulation (tDCS) during which small amounts of electric current are applied to the scalp to modulate the excitability of underlying neural populations (Nitsche and Paulus, 2000; Rosenkranz et al., 2000; Antal et al., 2004a; Paulus, 2011; Stagg and Nitsche, 2011; Jacobson et al., 2012). This is an appealing alternative because it can modulate the activity in relatively small regions of cortex without the influence of cortical reorganization as may happen with patients. In addition, a within-subjects design can be implemented. In tDCS the direction of current flow is determined by the placement of the anodal (+) and cathodal (−) electrode. Although it is a simplification, anodal tDCS has been associated with the depolarization of neurons and making them more likely to fire whereas cathodal tDCS has been associated with hyperpolarizing neurons and making them less likely to fire (Purpura and McMurtry, 1965). Although the mechanism of tDCS remains an area of active research, there is evidence to suggest that in the cortex tDCS modulates synaptic strength and likely stimulates pyramidal neurons and interneurons (Nitsche et al., 2005; Stagg and Nitsche, 2011). As a therapy, tDCS has shown some success in treating major depression (Fregni et al., 2006a,b; Brunoni et al., 2011), memory deficits in Parkinson’s disease (Boggio et al., 2006), memory deficits in Alzheimer’s disease (Boggio et al., 2009, 2011, 2012), aphasia (Baker et al., 2010; Kang et al., 2011; You et al., 2011), and as a recovery aid for stroke patients (Fregni et al., 2005b; Miniussi et al., 2008; Jo et al., 2009; Kang et al., 2009; Bolognini et al., 2011; Bueno et al., 2011). Despite these findings, less research has been done investigating the effects of tDCS on WM.

Several studies have used tDCS to investigate verbal WM. In these studies researchers have applied anodal tDCS to the left prefrontal cortex with the consistent finding that stimulation improved verbal WM performance using 2- and 3-back WM tasks (Fregni et al., 2005a; Ohn et al., 2008; Andrews et al., 2011; Mulquiney et al., 2011; Zaehle et al., 2011). These results also showed that cathodal stimulation of the left prefrontal cortex did not improve accuracy on the task. However, changes in cognitive abilities have not been tested with neuromodulation as thoroughly as with motor functions and in patient populations. Studies of tDCS in cognitive domains find a variable pattern of results and do not always match the predicted anodal-excitatory, cathodal-inhibitory effect (Jacobson et al., 2012).

Only two WM-tDCS studies that we know of have stimulated cortical regions other than the left prefrontal cortex. First, Berryhill et al. (2010) used tDCS to study PPC contributions to visual WM tested by recognition or recall. Healthy young adults who received cathodal tDCS to the right PPC (P4) were selectively impaired when making WM recognition judgments but performance on recall tasks remained intact (Berryhill et al., 2010). Anodal tDCS did not impair recognition WM. However, recently a second group found that *anodal* tDCS applied to the right PPC improved WM in a change detection WM recognition task, but cathodal tDCS had no effect on WM (Tseng et al., 2012, personal communication). In short, both studies found evidence for right PPC involvement in WM, specifically visual WM tested by recognition; however, the type of stimulation and the consequences of stimulation were inconsistent. There were several important differences between the studies that might have explained the different tDCS effects. First, there were important paradigmatic differences. The two WM tasks tested were quite different and they varied in task difficulty as well. The required amount of sustained attention and number of items was different between tasks. Another important difference between experiments was the difference in participants’ WM capacity, which was not measured in either of the previous studies. In this study, we report a different effect of tDCS depending on individual WM capacity. We reasoned that differential tDCS effects might be due to increased reliance on the PPC accompanying increases in task difficulty. However, this was only part of the story. To preview our results, tDCS applied to the PPC leads to different WM effects depending on WM task demand, but a second important factor is an individual’s WM capacity.

## Materials and Methods

### Experiment 1: PPC involvement in visual WM

The purpose of Experiment 1 was to determine the role of the right PPC in visual WM tasks. We directly compared performance in two previously tested WM recognition tasks (Berryhill et al., 2010; Tseng et al., 2012) that had confirmed functional PPC involvement in recognition WM but with inconsistent findings. In the first case (Berryhill et al., 2010), cathodal tDCS impaired WM performance and in the second case, anodal tDCS to the right PPC improved WM performance (Tseng et al., 2012, personal communication). Here, participants performed both WM tasks in a within-subjects design. A perfect replication of each of the previous findings would have required a complex pattern of results. Namely, anodal tDCS to the right PPC was expected to benefit the change detection WM task, but not the sequential WM task and cathodal tDCS to the right PPC was expected to disrupt the sequential WM task but not the change detection task. Although this prediction is based on the previous findings it struck us as unparsimonious because it required a tDCS (cathodal, anodal) by task (change detection, sequential presentation) crossover interaction. We thought it would be more likely that anodal or cathodal tDCS to the PPC would have uniform effects on WM performance in both tasks. For example, anodal stimulation should improve performance on both tasks or cathodal stimulation should inhibit performance on both tasks. This would be the case unless other task related factors were mediating the role of the PPC.

#### Participants

Twenty neurologically normal right-handed young adults (average age 23.25, SD 3.46, 12 females) participated. No participants were under the effects of neuroleptic, hypnotic, or seizure medications. No participant had a history of significant neurological or psychiatric disease or significant head injuries. All procedures were conducted in accordance with the University of Nevada Institutional Review Board. Participants were compensated $15/hour.

#### TDCS protocol

As in Berryhill et al. (2010) and Tseng et al. (2012), there were three tDCS testing sessions: anodal, cathodal, and sham (control condition). Sham stimulation incorporates 20 s of stimulation during the ramping up phase as in the actual stimulation conditions, however, after the 20 s stimulation ends. This has been shown to be an effective method for keeping participants blind to the condition (Gandiga et al., 2006). Conditions were administered on different days during 30-min testing sessions counterbalanced across participants. In all conditions one electrode was placed over the right parietal cortex at P4 (International 10-20 EEG system). The reference electrode was placed on the contra lateral cheek (Berryhill et al., 2010). In the anodal condition the anode was over P4 and in the cathodal condition the cathode was over P4. P4 was selected because it was used in both of the previously described PPC studies and would lead to closer replication of the methods used. P4 also was shown to influence WM recognition in previous studies. In the sham condition either the anode or cathode was placed over P4 in counterbalanced order. The order of stimulation conditions was counterbalanced across participants. Participants often took part in the study in consecutive days, however some gaps were longer. The gaps between sessions did not extend beyond a week between sessions. No participants reported any side effects which is consistent with other tDCS studies (Kessler et al., 2012).

Stimulation consisted of a single continuous direct current delivered by a battery-driven continuous stimulator (Eldith MagStim, GmbH, Ilmenau, Germany). Current was delivered through two 5 cm × 7 cm electrodes housed in saline-soaked sponges. During cathodal and anodal stimulation 1.5 mA current was applied for 10 min. Previous studies have found an effect of tDCS with 10 min of stimulation (Furubayashi et al., 2008; Berryhill et al., 2010; Andrews et al., 2011; Mulquiney et al., 2011; Antal et al., 2012; Berryhill and Jones, 2012; Kasashima et al., 2012). During sham stimulation participants received stimulation in which current lasted for 20 s at the start and end of the 10 min but no stimulation occurred in between. This gives participants the experience of feeling a minor tingling at most to have the appearance of stimulation. During stimulation participants performed practice trials of both procedures as to become familiar with each task. Immediately following the 10 min the electrodes were removed and the researchers left the room so that the participant could perform the task. Both experimental procedures were programmed using ePrime 2.0 (PST, Pittsburgh, USA). The experiment was conducted on Dell Optiplex 980 computer and stimuli were presented on a Dell 24″ monitor which participants sat 57 cm from. The University of Nevada Reno IRB approved all protocols.

#### Experimental tasks

##### Sequential presentation task

**Figure 1:**
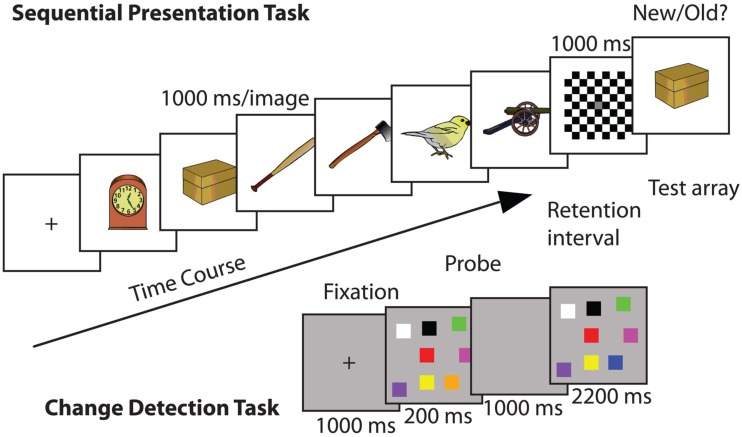
**Example trials of each of the WM tasks used in Experiment 1: (top) sequential presentation WM task: in each trial a series of images were presented and after a delay a probe image appeared: (bottom) change detection WM task in which a visual array was presented and after a delay a probe image appeared**. In both cases the response was to report whether the probe image was old or new.

Here, six visual stimuli were presented sequentially at fixation (1000 ms each; Berryhill and Olson, 2008a; Berryhill et al., 2010). The visual stimuli consisted of 72 colorized drawings of common objects (e.g., frog, arm; Rossion and Pourtois, 2004). The stimuli were approximately 20° × 10° of visual angle and they were presented on a uniform white background. A checkerboard mask (1000 ms) appeared after the sixth stimulus and then a seventh test stimulus appeared (until response). The test stimulus was one of the previous six 50% of the time (old) and a new stimulus 50% of the time (new). Participants made a new/old button response to indicate if the seventh test item was one of the first six (Figure 1).

##### Change detection task

The change detection WM task was similar to that used by Tseng et al. (2012). Each trial began with a central fixation cross (1000 ms). Next, eight randomly colored squares (3° × 3° of visual angle) appeared simultaneously at random locations presented against a medium gray background (200 ms), followed by a retention interval (1000 ms). The colored squares were equiluminant with the exception of black and white. The RGB values were as follows: yellow (255, 255, 0), white (255, 255, 255), teal (0, 210, 255), red (255, 0, 0), purple (156, 0, 255), pink (255, 0, 255), orange (255, 168, 0), green (0, 255, 0), blue (0, 0, 255), black (0, 0, 0), and aqua (0, 255, 216). The colored squares were created in Adobe Photoshop and only the hue changed between the squares. The luminance level remained the same. At test, the stimulus display reappeared (2200 ms) and participants had to make an old/new response indicating whether a single square had changed color (50% trials). The background color differences between the trial types helped to inform the participants of which type of trial would be next (Figure 1).

##### Digit span

We also administered tests of forward and backward digit WM span to each participant before the sham stimulation session. For each participant a combined score (forward span + backward span) was calculated as a measure of WM span. The digit span task is a useful measure of cognitive abilities. The digit span task is frequently used to measure cognitive capabilities (Parkinson et al., 1980; Conklin et al., 2000; Pisoni and Geers, 2000; Lefebvre et al., 2005).

#### Analysis

Here, we report the data using normalized difference indices (tDCS − sham/tDCS + sham) to minimize between-subject variability. Difference indices were used to normalize the effect of stimulation for each participant. These values were compared using a mixed model repeated measures ANOVA with the within-subject factors of task (sequential presentation, change detection) and tDCS condition (anodal, cathodal) and the between-subjects factor of WM span (high, low). Several other measures of WM performance accuracy were calculated [raw accuracy, corrected recognition (CR), WM capacity (Cowan’s *K*), and discrimination (*d*′)] with consistent findings across measures. All analyses were subject to Bonferroni correction.

#### Results

To demonstrate that there was no effect of tDCS unless WM capacity was considered, we conducted a repeated measures ANOVA including the within-subjects factors of WM task (sequential presentation, change detection) and the two stimulation difference indices (anodal, cathodal). As expected, there were no main effects of task or stimulation condition and no significant interactions (all *p*’s > 0.50). We anticipated this result, as this analysis failed to adequately account for the pattern in the data because it did not include a cognitive measure of WM capacity. We divided the participants into two groups based on their WM capacity. High and low WM capacity groups were defined by a median split on the combined forward and backward WM digit span scores. The high and low WM capacity groups had significantly different combined digit span scores (*M* low = 10.80 SD = 1.14, *M* high = 14.10 SD = 0.74, *t*_18_ = 7.71, *p* < 0.001). The forward digit span scores had a range of 5–9 and the backward digit span scores had a range of 4–7.

**Figure 2:**
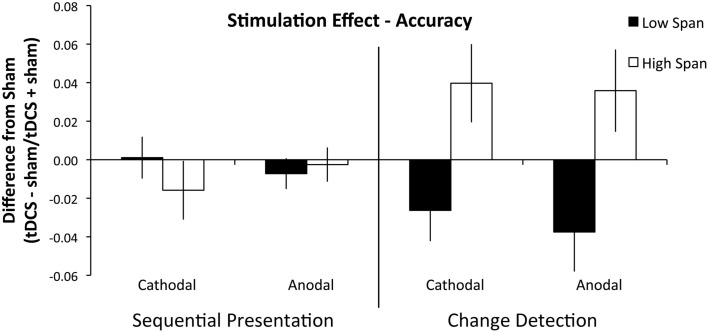
**Experiment 1 results**. Performance is plotted as a difference index using performance accuracy values (tDCS − sham/tDCS + sham). Values above 0 indicate superior performance in the tDCS condition; values below 0 indicate impaired performance after tDCS. The sequential task is presented on the left and the change detection task on the right. The low WM span group is plotted in black and the high WM span group is plotted in white. Error bars represent the SEM. There was a significant between-group effect of tDCS across stimulation condition and WM task.

A second repeated measures ANOVA on the difference indices of accuracy including the between-subjects factor of group found that there were no main effects of stimulation condition (*F*_1, 18_ = 0.096, *p* = 0.760, partial η^2^ = 0.005), or WM task (*F*_1, 18_ = 0.553, *p* = 0.467, partial η^2^ = 0.030). The main effect of WM capacity group was significant (*F*_1, 18_ = 5.685, *p* = 0.028, partial η^2^ = 0.240), such that the high WM capacity group received a benefit of tDCS and the low capacity group was impaired by tDCS; see Figure 2. Importantly, the interaction of WM capacity group and WM task was significant (*F*_1, 18_ = 9.648, *p* = 0.006, partial η^2^ = 0.349). The high WM capacity group received a global tDCS benefit and the low WM capacity group was globally impaired by tDCS, but this difference only emerged in the change detection task. To characterize the difficulty differences between the two tasks we compared performance in both tasks with *d*′ using a paired-samples *t*-test and found that performance on the sequential presentation task was significantly better than performance on the change detection task (*d*′ mean: sequential presentation task: 2.88, SD = 0.67, change detection task: 0.81, SD = 0.10, *t*_19_ = 10.58, *p* < 0.001, *r*^2^ = 0.85).

To investigate further, we conducted two independent samples *t*-tests on the difference indices of accuracy on the change detection task between WM capacity groups and found that the anodal (high WM capacity mean difference index = +3.6, low WM capacity mean difference index = −3.8: *t*_18_ = 2.612, *p* = 0.018, *r*^2^ = 0.13) and cathodal (high WM capacity mean difference index = +3.9, low WM capacity mean difference index = −2.6: *t*_18_ = 2.694, *p* = 0.015, *r*^2^ = 0.29) effect between WM capacity groups was significant. No other interactions approached significance (all *p*’s > 0.384). We also conducted two independent samples *t*-test on the sequential presentation task between WM capacity groups and found that the anodal (high WM capacity mean difference = −0.003, low WM capacity mean difference = −0.007: *t*_18_ = 0.954, *p* = 0.353, *r*^2^ = 0.05) and cathodal (high WM capacity mean difference = +0.021, low WM capacity mean difference = +0.001: *t*_18_ = 0.418, *p* = 0.681, *r*^2^ = 0.01) effect between WM capacity groups was not significant (Table 1).

**Table 1:** **Mean accuracy scores (SD) for all participants (total), the high WM capacity group (H), and the low WM capacity group (L)**.

	Sham			Anodal			Cathodal		
	Total	H	L	Total	H	L	Total	H	L
E1. Sequential Presentation	91 (0.05)	90 (0.04)	91 (0.06)	90 (0.06)	90 (0.05)	90 (0.06)	90 (0.08)	88 (0.07)	92 (0.09)
E1. Change Detection	63 (0.07)	63 (0.08)	64 (0.07)	64 (0.10)	68 (0.08)	60 (0.10)	64 (0.08)	68 (0.08)	61 (0.06)
E2. Set Size 4	82 (0.06)	84 (0.02)	82 (0.01)	83 (0.07)	84 (0.02)	81 (0.02)	81 (0.08)	83 (0.02)	79 (0.02)
E2. Set Size 6	69 (0.06)	70 (0.02)	68 (0.02)	71 (0.06)	73 (0.02)	69 (0.01)	69 (0.07)	70 (0.03)	68 (0.01)
E2. Set Size 8	62 (0.05)	62 (0.02)	62 (0.01)	66 (0.06)	68 (0.01)	64 (0.02)	65 (0.07)	67 (0.01)	63 (0.02)

*Rows 1 and 2 represent the tasks from Experiment 1 (E1) and rows 3–5 represent the set sizes in Experiment 2 (E2)*.

#### Discussion

Experiment 1 showed that the parietal contributions to WM may be quite different depending on participant’s WM capacity. High and low WM capacity groups responded in nearly equal and opposite directions to parietal tDCS. This finding replicated Tseng et al.’s report of anodal improvement of WM performance. However we only observed this effect *in the high WM capacity group*. We also observed improved WM performance after cathodal tDCS to the right PPC in the high WM capacity group. The Berryhill et al. data were also partially replicated, but only in the low WM capacity group, and only in the change detection WM task. In short, these data partially replicated Berryhill et al. (2010) and Tseng et al. (2012). Previous tDCS studies targeting parietal cortex reported a similar effect of anodal and cathodal stimulation. Here, WM performance in the high WM capacity group improved after either anodal and cathodal tDCS whereas performance in the low WM capacity group was impaired. Furthermore, the effect of tDCS on the change detection task performance was significantly greater than the effect on performance in the sequential presentation task.

In Experiment 1 there were significant differences in the two WM tasks. Neither the high nor low WM capacity group experienced a significant effect of tDCS on the sequential presentation task. This task was significantly easier and slower paced than the change detection task and it raises the possibility that the PPC was not recruited equally in each task. Additionally, in the sequential presentation task there may have been alternative strategies (e.g., verbal rehearsal of items) that activated other cortical regions to compensate for altered PPC function. A verbal rehearsal strategy would be impossible in the change detection task because of the fast presentation rate and the difficult-to-name aspect of the spatial configurations. However, performance on the change detection task was modulated by tDCS and WM capacity. The high WM capacity group benefited from anodal *and* cathodal tDCS suggesting that the PPC was differentially contributing to WM performance in low and high WM capacity groups.

### Experiment 2: Modulating task difficulty in WM change detection

There were several limitations in Experiment 1. First, there was a notable inter-task difficulty differential: the change detection task was significantly more difficult than the sequential presentation task. Second, high and low WM capacity individuals showed nearly equal and opposite effects of right PPC stimulation. Consequently, in Experiment 1 it was impossible to determine whether differences in WM performance were due to WM task demands or WM capacity. Experiment 2 addressed these confounds. We parametrically modulated task difficulty by varying the set size in the change detection WM task. We predicted that PPC involvement would increase with WM load as seen in previous fMRI research (Todd and Marois, 2004, 2005; Song and Jiang, 2006; Xu and Chun, 2006) and supported by our findings from Experiment 1. If the results in Experiment 1 were due to task difficulty, increasing WM task difficulty should place greater demands on relevant cortical structures such as the PPC and result in linear effects and improved WM performance.

#### Participants

Twenty-eight neurologically normal right-handed young adults (mean age 22.29, SD 3.05, 24 females) participated. Seven participants had also participated in Experiment 1. We conducted the same median split from Experiment 1 on the combined digit span scores for all participants. This allowed us to create a high WM capacity (mean = 14.07, SD = 1.59) and low WM capacity group (mean = 10.42, SD = 0.76). The range for the forward digit span was from 5 to 9 and the range of the backward digit span was 3–9.

#### Methods

Experiment 2 repeated the tDCS protocol and the change detection WM task described in Experiment 1 with one change. Additional set sizes (4, 6, and 8) were included to parametrically vary task difficulty. There were 100 trials of each set size pseudo randomly interleaved for a total of 300 trials. Anodal, cathodal, and sham conditions were used in a counterbalanced order across participants. The experimental task lasted ∼20 min.

#### Results

**Figure 3:**
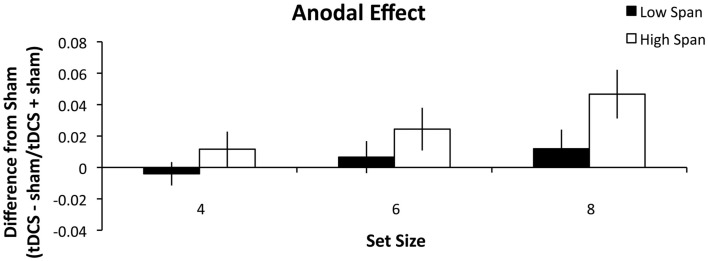
**Experiment 2 results**. The difference indices for anodal tDCS on WM accuracy. Error bars represent the SEM.

**Figure 4:**
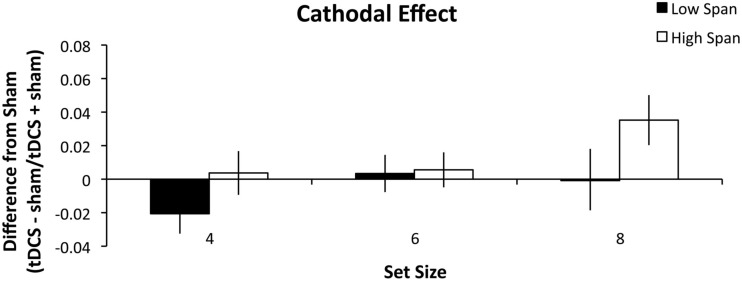
**Experiment 2 results**. The difference indices for cathodal tDCS on WM accuracy. Error bars represent the SEM.

As in Experiment 1, high and low WM capacity groups were defined by performing a median split on their combined forward and backward digit span scores. A repeated measures ANOVA was conducted analyzing the within-group factors of stimulation (anodal, cathodal), and set size (4, 6, and 8) and the between-group factors of WM capacity group (high, low). There was a main effect of stimulation condition (*F*_1, 26_ = 5.060, *p* = 0.033, partial η^2^ = 0.163) such that anodal stimulation (Figure 3) benefited WM performance more than cathodal stimulation (Figure 4). There was also a main effect of set size (*F*_2, 52_ = 4.375, *p* = 0.018, partial η^2^ = 0.144) such that tDCS effects followed a significant linear trend (*p* = 0.008, partial η^2^ = 0.240). Specifically, as set size increased, the effect of stimulation also increased. The within-subject contrast analysis on high WM capacity difference scores showed a linear trend for both anodal (*p* = 0.030, partial η^2^ = 0.314) and cathodal stimulation (*p* = 0.037, partial η^2^ = 0.294). It is possible that the cathodal effect in the high WM capacity group could best be explained by an exponential fit. However, paired-samples *t*-test indicated that there was no significant difference between mean *r*^2^ values as expressed by a linear (*M* = 0.534) versus exponential (*M* = 0.527) trend (*t*_13_ = 0.120, n.s.) Finally, the between-subject effect of group reached significance (*F*_1, 26_ = 5.097, *p* = 0.033, partial η^2^ = 0.164). The high WM capacity group showed a performance improvement following stimulation. Stimulation had a negligible effect on performance for the low WM capacity group. We conducted additional one-sample *t*-tests comparing the difference indices of the high WM capacity group from zero, or no change. The difference scores for the set size of 8 were significant for both the anodal (*t*_13_ = 3.303, *p* = 0.006, *r*^2^ = 0.46) and cathodal stimulation (*t*_13_ = 2.725, *p* = 0.017, *r*^2^ = 0.36). Pairwise comparisons of the 4 and 6 set size values were not significant (all *t*-values > 0.083). The same comparisons for the low WM capacity group were conducted and no measures reached significance. The cathodal difference score for a set size of four was the closest to significance (*t*_13_ = 1.802, *p* = 0.095, *r*^2^ = 0.20). None of the interactions reached significance (all *p*’s > 0.268).

#### Discussion

In Experiment 2, we investigated the role that task difficulty plays in PPC involvement in WM task performance. This experiment eliminated the task by difficulty confound in Experiment 1 by parametrically modulating set size to create three different levels of difficulty. Following Experiment 1, we predicted that the high WM capacity participants would benefit from tDCS and that the low WM capacity group would be impaired. We found that the high WM capacity group again benefited from either anodal or cathodal tDCS and that this benefit increased as task difficulty increased. However, here, the low WM capacity group was largely unaffected by tDCS except the decrease in performance seen following cathodal stimulation in the set size 4 condition. The increased benefit seen in performance following stimulation as difficulty increases reflects the strain put on the PPC by the task demands. This leads us to conclude that the PPC is needed more for recognition tasks that are more demanding than for those that are not. This is in support with previous research showing that PPC activity is greater with increasing WM loads (Todd and Marois, 2004; Song and Jiang, 2006).

### General discussion

Here we confirmed PPC involvement in WM using tDCS. In Experiment 1, we compared the effects of anodal and cathodal P4 tDCS on two different WM tasks: sequential presentation and change detection. Stimulation effects were greater in the change detection task. We also found that the direction of the tDCS effects depended on participants’ WM capacity. The low WM capacity group’s performance was generally impaired by tDCS whereas the high WM capacity group’s performance improved. Again, it was important to demonstrate that ignoring important group differences would obscure significant findings. Future research using tDCS should take this into account, as small sample sizes are common in tDCS studies making between-groups analyses under powered. In Experiment 2, we found that tDCS effects increased with WM task difficulty. As in Experiment 1, there were group differences. The high WM capacity group benefited from tDCS, and this effect was strongest in the anodal tDCS condition. Accordingly, we concluded that PPC involvement is greater in more difficult WM recognition tasks. These findings serve to resolve some of the discrepancy in the WM-tDCS literature by showing that tDCS to functionally involved regions can either improve or impair performance. The implications of these findings are discussed below.

#### PPC involvement in visual WM

The current findings are consistent with an interpretation we previously espoused called the *internal attention* model (Berryhill et al., 2011). Briefly, PPC contributions to WM can be described as strategically attending to items in WM and refreshing their representations. Accordingly, PPC involvement was predicted when the memoranda were difficult to verbalize, when attentional switching was compromised by a dual task paradigm, and when a passive WM strategy was adopted. This last prediction is thought to be associated with WM trials probed by recognition because participants may not engage an active verbal rehearsal strategy that is thought to draw more heavily on prefrontal involvement. The present WM paradigms probed WM using recognition. Yet, differential effects of PPC stimulation were noted in Experiment 1. This observation is consistent with our previous predictions because the change detection task met several of the criteria of the internal attention hypothesis: the stimuli were difficult to rehearse, making a deliberate verbal rehearsal strategy difficult. The change detection task may therefore be more reliant on attentional refreshing than the sequential presentation task. Furthermore, the slow pace of the sequential presentation task may not strain attentional resources as heavily as the faster-paced change detection task.

Previous research has shown that anodal tDCS to the right PPC, but not the left PPC, improves visual search and attentional skills (Bolognini et al., 2010). This was shown by improving visual search performance after tDCS and task training. The visual search findings help to explain our results as well. In the current study, anodal tDCS may not have only boosted attentional resources in the PPC allowing for better performance, but it also may have made visual processing more efficient. It is also possible that participants varied their strategy in the sequential presentation task and sometimes performed an active rehearsal strategy and other times relied on attentional refreshing. Averaging WM performance across trials would also show a smaller effect of tDCS than what was observed in the change detection task. Another factor that we previously predicted would increase PPC involvement was task difficulty. This prediction was born out in Experiment 2. We conclude that these data are consistent with a role for the PPC in the attentional refreshing process.

#### Group differences modulate tDCS effect size

Perhaps the most interesting finding here were the differences in the effect of tDCS to the PPC on low and high WM capacity groups. The high WM capacity group revealed a greater benefit of tDCS across WM tasks and stimulation condition. However, the low WM capacity group did not see a uniform stimulation effect across both experiments. In Experiment 1 the low WM capacity group was uniformly impaired by tDCS. This pattern of nearly equal and opposite effects in high and low WM capacity groups may explain why previous groups have had difficulty identifying any effect of tDCS. In Experiment 2, there was no effect of tDCS in the low WM capacity group. Previously we reported that less educated older adults did not benefit from frontal lobe tDCS but better educated adults benefited (Berryhill and Jones, 2012). Experiment 2 replicated the finding that high WM capacity predicted a larger benefit of tDCS whereas low WM capacity showed no improvement. We suspect that the differences in digit span score and education level both are reflecting the same underlying mechanism. To our knowledge there are only two other studies incorporating measures of group differences. In one case a motor learning task showed that the effect of tDCS to the motor cortex varied according to a participant’s genotype for brain-derived neurotrophic factor (BDNF; Cheeran et al., 2008). In the second, in an emotional stimulus categorization task, tDCS to the dorsolateral prefrontal cortex had a greater effect on introverts than extraverts (Pena-Gomez et al., 2011). Future studies will be needed to identify the relevant factors influencing the magnitude of tDCS effects.

Other researchers have found important differences in WM strategy across individuals with different WM capacities (e.g., Cokely et al., 2006; Imbo and Vandierendonck, 2007; Bailey et al., 2008; Baldwin and Reagan, 2009; Unsworth and Spillers, 2010). Low WM span individuals are less able to ignore distracters (Unsworth, 2007), rely on context to recall items, and have fewer attentional resources (Conway and Engle, 1996; Kane et al., 2001; Unsworth and Spillers, 2010). Recent research has shown that high WM capacity participants adopted more efficient strategies in a category naming task compared to low WM capacity participants (Schelble et al., 2012). Importantly, however, when instructed to use the same strategy as the high WM capacity participants the low WM capacity participants performed just as well. This suggests that it is not a fundamental inability but rather a miscalculation that can be remedied through training. Another recent WM study found that participants used different strategies based on the demands of the WM task (Sandrini et al., 2012). In a series of *n*-back tasks, participants employed different strategies for 1-back, when compared to 2- or 3-back tasks. These authors conclude that the 1-back tasks can rely on stimulus familiarity because the task is to identify repetitions whereas 2- or 3-back tasks may require recollection to overcome the presence of intervening stimuli. Further research is underway to examine whether the differences we observed can be explained by differences in WM strategy. Particularly given the safety and affordability of tDCS, it will be important to define with some confidence who, when, and how individuals will benefit from tDCS.

#### Mechanisms of tDCS

Apart from WM strategy, tDCS may have different effects on participants because of differences in their biology (morphological and genetic), which remain poorly understood. Animal research involving tDCS found that anodal tDCS increased neuronal activity and cathodal tDCS decreased neuronal activity (Purpura and McMurtry, 1965). However, within deeper layers of cortex, the opposite effect was seen such that anodal stimulation deactivated neurons and cathodal stimulation activated them. This suggested that neuronal orientation is important to understanding the effect of tDCS (Purpura and McMurtry, 1965). Within the cortex, tDCS modulates synaptic strength and likely stimulates neurons in the cortex, pyramidal neurons, and interneurons (Stagg and Nitsche, 2011). Several neuromodulators such as GABA (Stagg et al., 2009), Na^+^ and Ca^2+^ channel blockers (Nitsche et al., 2004), l-DOPA (Kuo et al., 2008), and the D_2_ receptor agonists (Nitsche et al., 2006; Monte-Silva et al., 2009) also have an effect on increasing and/or decreasing the effects of tDCS stimulation (for more see Stagg and Nitsche, 2011). Some progress in linking DNA genotypes with cognitive performance is underway. Different genotypes reflect differences in the biology, such as neurotransmitter level or ion channel subtypes, that may affect the influence of tDCS. The catechol-*O*-methyltransferase (*COMT*) gene codes for an enzyme that metabolizes catecholamines and it is particularly important for metabolizing prefrontal dopamine. A single point mutation in the *COMT* gene (val158met) is associated with differences in cognitive abilities (de Frias et al., 2004; Bruder et al., 2005; Bertolino et al., 2006; Aguilera et al., 2008; Stokes et al., 2011; Buckert et al., 2012). There is also some evidence that *COMT* genotype has a significant effect on the volume of gray matter and parietal lobe activity (Dumontheil et al., 2011). Consequently, *COMT* genotype may play a role in determining how participants will respond to tDCS, or whether they have a low or high WM capacity. This complex story will require collaboration between neuroscientists focusing on all of these levels to enable accurate prediction of the effect of tDCS.

There are also discrepancies between studies in the tDCS literature that deserve mention. The relationship between stimulation condition and its effects are not fully understood. The assumption with tDCS in studies of cognition is that there is an excitatory effect of anodal current and an inhibitory effect of cathodal current. As shown in a recent meta-analysis this is commonly observed in studies of motor cortex stimulation, but this pattern is only rarely seen in studies of cognition (Jacobson et al., 2012). One explanation for this are that cognitive abilities are more active than motor functions during stimulation as participants are generally not moving but still have active WM. Motor behavior is not voluntarily activated during stimulation whereas WM is constantly being updated. Measures of cognitive task performance may also be more susceptible to external noise than measures of motor task performance. This may be because motor tasks are generally measured with motor evoked potentials whereas cognitive performance is measured by a variety of ways such as reaction time, accuracy, and neuroimaging (e.g., fMRI, ERP, and MEG; further reviewed in Jacobson et al., 2012). Some examples of studies of cognitive functions that do not follow the anodal-excitatory, cathodal-inhibitory pattern are picture naming (Monti et al., 2008), risk-taking (Boggio et al., 2010), and reaction time on a visual Sternberg task (Marshall et al., 2005). Also, cathodal tDCS may not be decreasing neural excitability, but it may be reducing competition between neurons (Antal et al., 2004b). Another explanation is that cathodal tDCS to the right PPC acts as a noise filter and helps to suppress distractors and boost performance (Weiss and Lavidor, 2012). This predicts a greater benefit of tDCS at greater set sizes, consistent with our finding that there was a greater benefit at set size 8 than 4 or 6.

#### Limitations and open questions

One limitation of the present analysis is that we conducted a median split based on the combined digit span scores. Median splits eliminate the continuous nature of the digit span variable. Future individual differences investigations will be needed to more precisely assess the relationship between WM capacity and parietal lobe involvement in WM tasks. These findings show that at the coarser group level there are differences. We speculate that the nature of these differences may be reflecting different strategies in accomplishing WM tasks. Another criticism is that we assessed WM capacity based on digit span scores. It has been suggested that the digit span measure does not correlate as well as complex WM span tasks with fluid intelligence (Chein et al., 2011). Complex WM span tasks require attention to shift away from the to-be-remembered items to perform a second distracter task. This is a more realistic representation of the way WM operates in everyday life. To address this concern we have begun to collect Operation span measures (Turner and Engle, 1989; Unsworth and Engle, 2005) from our participants in addition to forward and backward digit span. Operation span task requires participants to remember a series of words interleaved with distracter arithmetic equations. We conducted this measure on 16 of the 28 participants. Analyses conducted based on these scores were consistent with groups defined by digit span. To date, people who have been tested on both measures reveal the same pattern of data. This provides some assurance that dividing groups based on digit span is likely to produce similar results.

A second limitation of this work is that tDCS cannot claim to focally stimulate a particular aspect of the PPC. We were careful to use this overly general term even though the PPC is clearly composed of multiple functional subsections – e.g., the superior parietal lobule, supramarginal gyrus, and angular gyrus. This problem of identifying the site of tDCS stimulation is currently being addressed through the application of cortical modeling (Datta et al., 2009a, 2011; Mendonca et al., 2011). These modeling data can provide considerable insight to the unintuitive spread of current through the cortex. For our purposes, the between-subjects findings are important because the same electrode montages were applied to all participants. Consequently, even though we cannot state with precision the boundaries of stimulation, we can state that there were differential effects as a function of WM capacity. In the future the development of High Density tDCS (HD-tDCS) techniques will permit greater specificity in estimating the extent and specificity of cortical stimulation (Datta et al., 2009b; Diaz et al., 2009; Dmochowski et al., 2011). The combination of cortical modeling and HD-tDCS will supplement the researcher’s armamentarium and provide an effective and safe investigational tool to probe brain structure–function relationships.

## Conflict of Interest Statement

The authors declare that the research was conducted in the absence of any commercial or financial relationships that could be construed as a potential conflict of interest.
